# Supplementary material for: Genomic and metabolic insights into the first host-associated isolate of Psychrilyobacter
Source: Microbiol Spectr. 2023 Sep 27;11(5):e03990-22. doi: 10.1128/spectrum.03990-22 (PMC10580919; doi:10.1128/spectrum.03990-22)
Supplement: Supplemental text — Supplemental materials and methods. [file spectrum.03990-22-s0001.docx]

**Materials and methods**

**16S rRNA gene library construction, sequencing and data processing**

Briefly, total DNA of intestinal content was extracted using a FastDNA™ SPIN Kit according to the manufacturer’s instructions. The V1-V9 region of the bacterial 16S ribosomal RNA gene was amplified by PCR using primers of 27F 5’-AGRGTTYGATYMTGGCTCAG-3’ and 1492R 5’-RGYTACCTTGTTACGACTT-3’, where the barcode is an eight-base sequence unique to each sample. PCRs were performed in triplicate. The PCR product was detected using 2% agarose gel electrophoresis and purified using the AxyPrep DNA Gel Extraction Kit (Axygen Biosciences, Union City, CA, U.S.) according to the manufacturer’s instructions.

SMRTbell libraries were prepared from 16S rRNA gene PCR products by blunt ligation according to the manufacturer’s instructions (Pacific Biosciences). Purified SMRTbell libraries from the Zymo and HMP mock communities were sequenced on dedicated PacBio Sequel II 8M cells using the Sequencing Kit 2.0 chemistry. Purified SMRTbell libraries from the pooled and barcoded samples were sequenced on a single PacBio Sequel II cell. PacBio sequencing was performed at Shanghai Biozeron Technology Co., Ltd. (Shanghai, China).

Raw reads were processed through SMRT Portal to filter sequences for length (<800 or >2500 bp) and quality. OTUs (operational taxonomic units) were clustered with a 98.65% similarity cutoff (1) using UPARSE (version 7.1, http://drive5.com/uparse/), and chimeric sequences were identified and removed using UCHIME (version 4.1, http://drive5.com/uchime/uchime_download.html). The phylogenetic affiliation of each 16S rRNA gene sequence was analyzed by RDP Classifier (http://rdp.cme.msu.edu/) against the SILVA 16S rRNA gene database (SSU132) using a confidence threshold of 70%.

**Cell morphology observation and physiological and biochemical identification**

For the transmission electron microscopy (TEM), the fresh bacterial cell suspension was spread on copper grids and stained with 2% PTA (phosphotungstic acid). The cell morphology was observed under 120 KV with an electron microscope (JEM-1230, JEOL).

The growth temperature was determined by placing the streaked MA plates in incubators at different temperatures (4, 10, 12, 15, 28, 33, 37, and 40°C) for 72 h to observe the growth of the tested strain. The pH and salinity were determined by setting different pH gradients from 3 to 12 with an interval of 1 and different salinity gradients from 0 to 12% NaCl (w/v) with an interval of 1 for 96 h to observe the growth curves. Diverse algae-derived carbohydrates, including glucose, galactose, xylose, fructose, mannitol, trehalose, agar and alginate, were used as the only carbon source to test the capability of carbohydrate utilization. These carbohydrates were added to the artificial seawater to reach 1 g/L in 50 ml anaerobic flasks and then statically cultivated and observed through growth curves and cell counts.

To measure the fatty acid composition of strain B1, the cell biomass of strain B1 was collected from MA plates after 48 h of growth. Fatty acids were extracted from the biomass following four steps: saponification, methylation, extraction and alkali washing (2). Finally, fatty acids were measured by gas chromatographic analysis according to the midi system.

**Strain B1 cultivation in different fermenting mediums**

To determine the fermentation products of different subtracts, four different substrates were used as the only carbon sources, including tryptone (5 g/L, OXOID, Hampshire, England), glucose (5 g/L, XILONG SCIENTIFIC, Guangdong, China), manntiol (5g/L, Solarbio, Beijing, China) and trehalose (5 g/L, Solarbio, Beijing, China) as the sole carbon source, respectively, resazurin (1 g/L, Solarbio, Beijing, China) as an oxygen indicator, and L-cysteine hydrochloride (0.5 g/L, Macklin, Shanghai, China) as a reducing agent in the artificial seawater medium under anaerobic conditions (3).

First, the strain B1 was cultured in anaerobic 2216E marine broth at 15^o^C for 3 days to be as the seed liquid. Second, 1 mL of the seed was centrifugate at 8000 rpm for 5 min to remove the supernatant, then 1 mL of sterile artificial seawater medium was add to resuspend the precipitate. Third, 200 μL of the resuspended seed liquid was injected into each 20 mL medium with different substrates, and the original OD_600_ values are approximate 0.02±0.005. After 5 days’ cultivation, the OD_600_ value in each condition are 0.2115±0.0001 (tryptone), 0.0699±0.019 (Glucose), 0.0568±0.0057 (manntiol), 0.0577±0.0085 (trehalose), respectively. There are three biological replicates for each condition, and the condition of artificial seawater medium without carbon source is the control.

**SCFAs extraction**

The low-molecular-weight organic acids fermented by strain B1 were extracted using 100 mM H_2_SO_4_ solution, and the volume ratio of each fermented product to the H_2_SO_4_ solution is 19:1. After centrifugation at 8000 rpm for 5 min, 1mL of the supernatant was filtered through 0.22 μm membrane, and the filtrate was injected into the bottle for HPLC test.

**High-performance liquid chromatography (HPLC) test**

Chromatographic experiments were performed on high-performance liquid chromatograph HPLC Elite Waters HPLC Alliance e2695, equipped with 2414 R1 Detector, column thermostat, refractive index detector and empower software. The HPLC separation was carried out on Aminex® HPX-87H Ion Exclusion Column (300 mm × 7.8 mm) column. The mobile phase consisted of 0.005 M sulphuric acid with a flow rate of 0.3 mL/min. All samples before injection were filtered through 0.45 μm. The volume of the injected sample was 10 μL. Formic acid, acetic acid, propionic acid, and butyric acid standards were utilized to identify and determine their concentration in the samples. For this purpose, serial dilutions of standards were prepared according to the values of the areas under the curve of the fermentation samples.

**Results**

**HPLC plots and quantitative results**


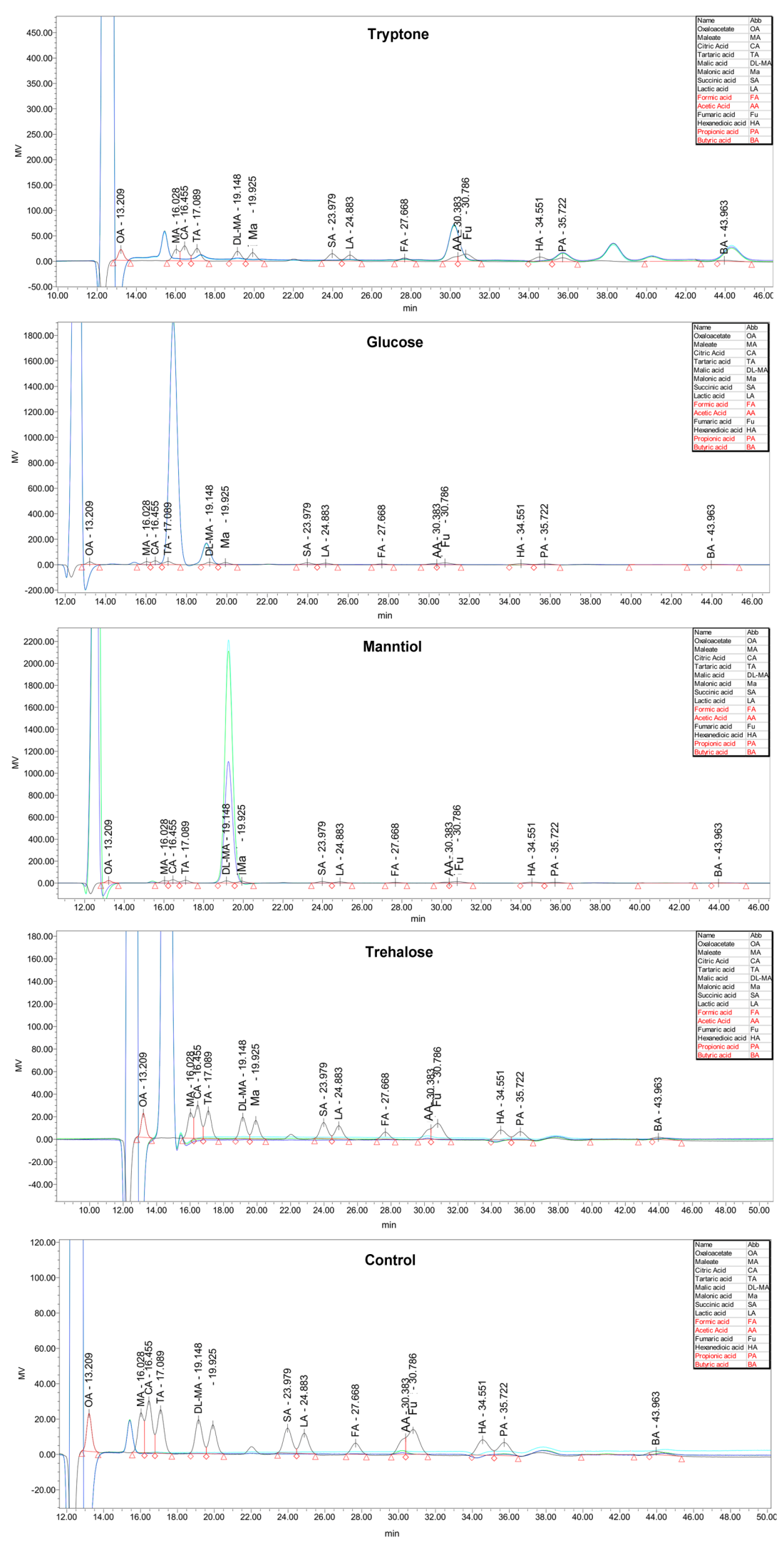


**Peak plot of fermentation products of strain B1 cultured in different conditions**

The horizontal and verticle axes represent the time and volume of mobile phase, respectively. The peaks with black color indicate the standards, and the abbreviations of their names and peak time are shown above the corresponding peaks. The full names and abbreviations are shown on the top-right of each plot.


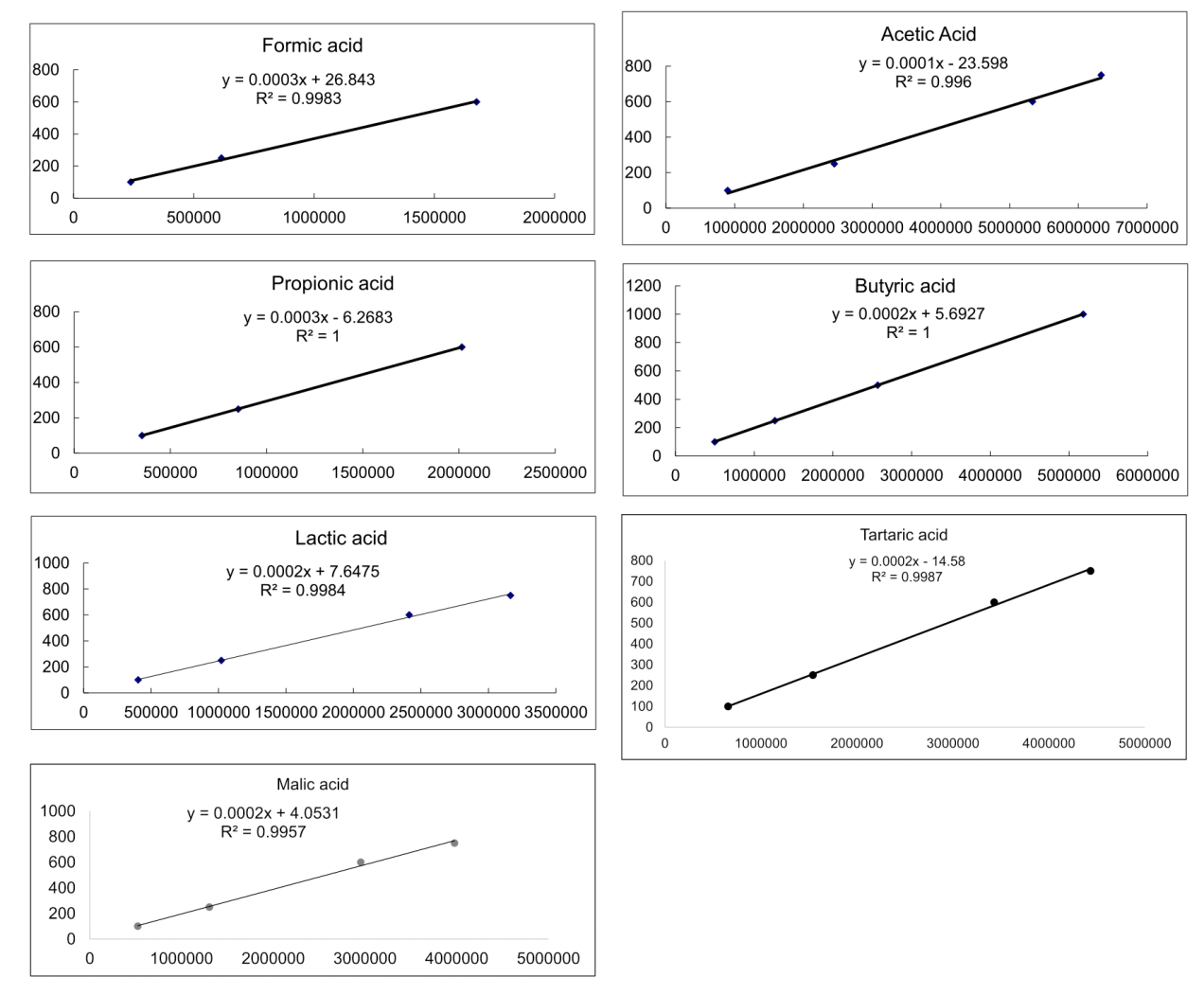


**The standard curves of the detected organic acids in this study**

The horizontal and verticle axes represent the peak volume and concentrations (mg/L), respectively.

**The quantitative results of the fermentation products of strain B1 cultured in different conditions**

| **Organic acid** | **Condition** | **Biological**  **replicate** | **Maintain time (min)** | **Area** | **Content**  **(mg/L)** |
| --- | --- | --- | --- | --- | --- |
| **Lactic acid** | Tryptone | 1 | 24.81 | 33009.36 | 14.25 |
|  |  | 2 | 24.84 | 27312.76 | 13.11 |
|  |  | 3 | 24.84 | 10424.25 | 9.73 |
| **Formic acid** | Tryptone | 1 | 27.67 | 41121.27 | 39.18 |
|  |  | 2 | 27.65 | 42829.78 | 39.69 |
|  |  | 3 | 27.66 | 58531.37 | 44.40 |
| **Acetic acid** | Tryptone | 1 | 30.19 | 2656086.00 | 242.01 |
|  |  | 2 | 30.19 | 2623902.00 | 238.79 |
|  |  | 3 | 30.19 | 2755940.00 | 252.00 |
| **Propionic acid** | Tryptone | 1 | 35.73 | 834427.80 | 244.06 |
|  |  | 2 | 35.73 | 829172.90 | 242.48 |
|  |  | 3 | 35.74 | 846925.20 | 247.81 |
| **Butyric acid** | Tryptone | 1 | 44.32 | 1495335.00 | 304.76 |
|  |  | 2 | 44.33 | 1475495.00 | 300.79 |
|  |  | 3 | 44.33 | 1798764.00 | 365.45 |
| **Tartaric acid** | Glucose | 1 | 17.34 | 52350027.00 | 10455.43 |
|  |  | 2 | 17.34 | 52825056.00 | 10550.43 |
|  |  | 3 | 17.34 | 53022202.00 | 10589.86 |
|  | Trehalose | 1 | 16.88 | 597950.00 | 105.01 |
|  |  | 2 | 16.88 | 860310.80 | 157.48 |
|  |  | 3 | 16.83 | 108985.60 | 7.22 |
| **Malic acid** | Glucose | 1 | 18.99 | 5026303.00 | 1009.31 |
|  |  | 2 | 18.99 | 4789708.00 | 961.99 |
|  |  | 3 | 18.99 | 4577231.00 | 919.50 |
|  | Manntiol | 1 | 19.25 | 30783611.00 | 6160.78 |
|  |  | 2 | 19.25 | 59213538.00 | 11846.76 |
|  |  | 3 | 19.26 | 61661540.00 | 12336.36 |

**References**

1. Edgar RC. 2013. UPARSE: highly accurate OTU sequences from microbial amplicon reads. Nat Methods 10:996-8.

2. Liu M, Huang Z, Zhao Q, Shao Z. 2019. Cohaesibacter intestini sp. nov., isolated from the intestine of abalone, Haliotis discus hannai. International Journal of Systematic and Evolutionary Microbiology 69:3202-3206.

3. Takai K IF, Nakagawa S, Hirayama H, Nunoura T 2003. Isolation and phylogenetic diversity of members of previously uncultivated epsilon-Proteobacteria in deep-sea hydrothermal fields. . FEMS Microbiol Lett 218:167–174.
